# From Awareness to Action: Pathways to Equity in Pain Management

**DOI:** 10.1089/heq.2023.0179

**Published:** 2023-08-23

**Authors:** Monica L. Wang, Olivia Jacobs

**Affiliations:** ^1^ Department of Community Health Sciences, Boston University School of Public Health, Boston, Massachusetts, USA.; ^2^ Office of Narrative, Boston University Center for Antiracist Research, Boston, Massachusetts, USA.; ^3^ Department of Health Policy and Management, Harvard T.H. Chan School of Public Health, Boston, Massachusetts, USA.; ^4^ American and New England Studies Program, Boston University, Boston, Massachusetts, USA.

**Keywords:** pain management, disparities, equity

## Abstract

This commentary calls for a shift in the identification, analysis, and treatment of disparities in pain management. We provide context and research that summarize social and structural determinants that contribute to disparities across multiple levels of the pain management continuum. Informed by the evidence, we provide guideposts for mitigating disparities in the assessment, diagnosis, and care provided to those experiencing pain, with a focus on highlighting the specific needs of marginalized communities and the importance of culturally appropriate and context-specific approaches to pain management. This commentary informs efforts to promote equity by identifying areas of concern, guiding interventions, and advocating for policies that aim to eliminate disparities in pain treatment. Researchers, health care providers, and organizations can collectively work to provide equitable culturally sensitive pain management and improve overall patient outcomes.

Despite pain being a universal human experience, disparities in pain assessment and treatment by race, ethnicity, gender, socioeconomic status, and other sociodemographic factors are well documented. A study of cancer patients living in the United States found that physicians underestimated pain severity among 74% of Black patients and 64% of Hispanic patients.^1^ Patients of color, especially Black patients, are less likely to be referred to pain specialists and less likely to be prescribed opioids less often than White patients who undergo similar surgical procedures.^2^

Women (defined as those who self-identify as women), despite experiencing and reporting more severe pain than men, receive less intensive and less clinically effective treatment for their pain.^3^ In a study of 981 patients, women with pain were less likely to receive analgesia and 10% less likely to receive opiates than men.^4^ Men are also more frequently prescribed opioids and in greater quantities than women, with the latter more likely to receive nonmedical recommendations for pain alleviation.^5^

Disparities in pain management are also observed across socioeconomic status, health care coverage and access, and geographic location. Although low socioeconomic status patients report higher postoperative pain levels, they receive fewer opioid prescriptions than higher socioeconomic status patients, according to research.^6^ Individuals living in rural areas, under-resourced communities, or health care deserts have less access to pain clinics or health care facilities.^6^ Those with less financial resources and lack of health insurance coverage and access also have limited access to nonpharmacological approaches to treat pain, which can further exacerbate disparities in pain management outcomes.^5^

Quantifying and treating pain can be challenging for a variety of factors, including the *subjective multidimensional experience* that encompasses physical, emotional, cognitive, and social aspects of pain; *lack of specific biomarkers* to measure pain (and thus reliance on self-report to assess severity and progression); *variability in pain tolerance*, which can be influenced by individual preference, age, psychological state, cultural backgrounds, and personal beliefs; and *communication barriers* (e.g., populations such as individuals with language barriers or limited health literacy, those with cognitive impairments, and young children are limited in their ability to communicate their pain effectively).

Given the subjective nature and complexity of pain, there is no universally accepted or standardized set of protocols to assess and diagnose pain. A variety of instruments have been developed to assess pain, including self-report scales, behavioral observations, and clinical examinations.^7^ However, many assessment tools lack standardization, have not been validated among or tailored for a variety of populations; thus, the current tools available to and used by health care professionals may contribute to disparities in pain assessment and management.^8^

## Guiding Principles to Target Disparities in Pain Management

A number of strategies can be used to enhance equity in pain management and improve overall patient outcomes through health care delivery, research, policy, and advocacy.

1. ***Improve and standardize pain assessment guidelines and protocols to assess multiple dimensions of pain and train providers accordingly.*** Standardized assessment tools such as the Numerical Rating Scales, Verbal Rating Scales, and Visual Analog Scales have demonstrated greater reliability in quantifying pain across language and cultural barriers.^9^ Comprehensive assessments include inquiring about physical, emotional, cognitive, and social dimensions of pain, assessing the severity, location, duration of pain, and applying perspective-taking and patient-centered communication. In a trial of 436 health care providers, those who received a tailored perspective-taking intervention were 85% less likely to exhibit pain treatment bias against Black patients and 76% less likely to exhibit pain treatment bias against patients with low socioeconomic status.^10^ Asking open-ended questions, avoiding use of judgment-based statements, and simplifying medical jargon can also facilitate better patient–provider communication and enhance outcomes.^11^
2. ***Tailor pain management approaches and contextualize care for diverse populations.*** Cultural beliefs and language can significantly impact how patients experience and report pain, and cultural values influence how patients make health care decisions, including pain treatment.^11^ Demonstrating respect for how patients may express pain differently and may prefer different approaches to pain management can facilitate more equitable pain management outcomes. When asking about pain, it may be more informative to use specific reference points such as asking patients to describe their current pain compared with previous experiences, or how their pain impacts daily living activities, rather than solely relying on a numerical scale.^12^ Providers can also inquire if there are any cultural restrictions regarding treatment plans that providers should be aware of.
3. ***Promote shared provider–patient decision-making and support patients as part of a larger community.*** Shared decision-making requires mutual recognition and respect for each party's expertise and experiences. To collaboratively explore treatment options and reach decisions that align with patient values and preferences, providers should provide comprehensive information, including potential benefits, risks, and side effects of different treatment options while respecting patient autonomy. Patients should consider the provider's expertise and guidance when making decisions. Nonprescription approaches that can be used in isolation or to complement other treatment include exercise, relaxation techniques, physical therapy, osteopathic interventions, and cognitive behavioral or massage therapy.^13^ By discussing different options (with an emphasis on approaches available and accessible to patients and their specific circumstances), providers can increase patient awareness of the spectrum of treatment plans, promote patient autonomy, and enhance adherence to care.^14^ Ongoing communication and reassessment is critical so that treatment is responsive to changes in patient's condition or preferences. When possible and relevant, providers should work to include key individuals such as family members in developing care strategies to mitigate risk and discomfort at home and connect patients with available support systems and community resources.
4. ***Invest in and support intervention research to inform evidence-based practices.*** Research that develops and evaluates interventions in collaboration with patients to reduce disparities in pain management is critical to inform change in practice and enhance patient care.^15^ To maximize impact and relevance to all patients, investigators should recruit diverse populations in their study design (with particular emphasis on representation of groups shown to have experienced inadequate pain management) and develop intervention approaches and evaluation methods in collaboration with key stakeholders, such as patients and patient advocates, community members, and health providers.
5. ***Advocate for policies that promote better access and care in pain management.*** Changes in the health care setting alone will not eliminate disparities in pain management. Broader structural and systemic factors need to be addressed, such as advocating for insurance coverage of evidence-based pain treatment, improving access to comprehensive pain management (e.g., access to pain specialists, multidisciplinary clinics, and nonpharmacological treatment options), investing in additional clinics and telehealth services and support in rural areas and health care deserts, and supporting research funding in pain management disparities.

Reducing pain management disparities requires a multifaceted approach involving various stakeholders, including health care professionals, researchers, policymakers, and patient advocates. By improving and standardizing pain assessment tools and protocols, investing in health equity intervention research, advocating for policy change, and aligning efforts across sectors, we can collectively work toward a patient-centered approach that is equitable and effective for all.
